# Gilles de la Tourette syndrome is not linked to contactin-associated protein receptor 2 antibodies

**DOI:** 10.1186/s13041-015-0154-6

**Published:** 2015-10-13

**Authors:** Kurt-Wolfram Sühs, Thomas Skripuletz, Refik Pul, Sascha Alvermann, Philipp Schwenkenbecher, Martin Stangel, Kirsten Müller-Vahl

**Affiliations:** Klinik für Neurologie, Medizinische Hochschule Hannover, Carl-Neuberg Str. 1, 30625 Hannover, Germany; Klinik für Psychiatrie, Sozialpsychiatrie und Psychotherapie, Medizinische Hochschule Hannover, Carl-Neuberg Str. 1, 30625 Hannover, Germany

**Keywords:** Tourette syndrome, Antineuronal antibodies, CASPR2, NMDAR, Tic

## Abstract

**Background:**

In Gilles de la Tourette syndrome (GTS) an immunopathogenic influence of autoantibodies is suspected. In familial GTS a disruption of the contactin-associated protein 2 gene (CNTNAP2), coding for the contactin-associated protein 2 (CASPR2), has been reported. Autoantibodies against CASPR2 are associated with other movement disorders like Morvan’s syndrome. In addition, positive oligoclonal bands (OCB) in cerebrospinal fluid (CSF) have been found in more than a third of GTS patients, indicating a pathological intrathecal immunoglobulin synthesis. These findings drove the hypothesis that CASPR2 antibodies are involved in GTS.

**Methods:**

In this cross sectional study, 51 patients with GTS were examined for CASPR2 and other autoantibodies. We used indirect immunofluorescence or enzyme-linked visualization in cell-based assays on tissue sections from cerebellum (rat and monkey), hippocampus (rat), and immunoblots for the detection of specific or any other autoantibodies.

**Results:**

Serum samples from 51 GTS patients, mean age 35.0 ± 13.1 y, were analyzed. In none of the 51 GTS sera CASPR2 antibodies were detectable. Neither had we found any other specific autoantibodies (LGI1, NMDAR, AMPA1, AMPA/2 or GABAB1/B2). An anti-nuclear pattern of immunoreactivity was observed in 7/51 (14 %) samples. In these patients an immunoblot analysis was used to rule out antibodies directed against well-defined intracellular target antigens. A specific anti-neuronal binding pattern could not be seen in any of the tissue sections.

**Conclusions:**

The results negate that CASPR2 antibodies play a role in the pathogenesis of Tourette syndrome and do not support the assumption that anti-neuronal antibodies are involved.

## Findings

### Introduction

Gilles de la Tourette syndrome is a chronic neuro-psychiatric disorder with an estimated prevalence rate of about 0.6–1 % [1]. It is thought that pathophysiologically both genetic vulnerability and environmental factors – including immunological changes - are involved. Supporting an immunopathogenic influence, elevated concentrations of Tumor necrosis factor alpha (TNF-α) and Interleukin 12 (IL-12) have been detected in patients with GTS [2]. In addition, positive oligoclonal bands in the cerebrospinal fluid have been found in 38 % of GTS patients [3]. This strongly suggests a pathological intrathecal immunoglobulin synthesis in GTS, because positive OCBs are found in only 3 % of the general population. However, the role of autoantibodies in GTS remains unclear, since contradictory results have been found [4].

In the last decade, several antibodies targeting neuronal surface proteins (especially ion channels) have been identified to be causative in different neurological disorders including idiopathic limbic encephalitis (LE) and Morvan’s syndrome [5]. For example in LE AMPA receptor antibodies (AMPA 1 and AMPA 2), which are directed against the GluA1 and GluA2 subunits of AMPA receptors, can be found. In Morvan’s syndrome, characterized by peripheral nerve hyperexcitability, an association with the contactin-associated protein 2 (CASPR2) has been demonstrated [6]. Accordingly, clinical improvement following immunotherapy has been reported [7]. In addition, CASPR2 is a known genetic risk factor of autism and has been suggested to play a role in several other neurodevelopmental disorders including ADHD and OCD [8]. CASPR2, expressed in juxtaparanodal regions of myelinated axons prominently in the brain, is linked to voltage gated potassium channels (VGKC) [9]. It is encoded by the contactin-associated protein 2 gene (CNTNAP2). Most interestingly, a disruption of the CNTNAP2 gene by chromosome insertion has been found in a GTS family in both the affected father and two affected children. The authors speculated that the disruption leads to a disturbed distribution of K^+^ channels causing unwanted movements like tics [10]. So far, only one other family - without GTS - has been described with a disrupted CNTNAP2 gene [11]. This observation led to the conclusion that not the disruption of the CNTNAP2 gene, but a dysfunction of the ion channel by CASPR2 antibodies might be causative in GTS. The aim of this study was to investigate for the first time CASPR2 antibodies in sera of a large group of adult patients with GTS.

### Methods

In this study, we included 51 consecutive adult patients with GTS according to DSM-IV-TR confirmed by one of the authors (KMV). All patients were recruited from the Tourette’s outpatient clinic at the Hannover Medical School. Blood samples were collected after approval by the ethics committee of the Hannover Medical School. Patients with autoimmune diseases of the CNS were not eligible to participate. All patients gave their written informed consent before entering the study.

From each patient 7.5 mL blood were drawn, centrifuged at 3500 rpm for 15 min, and serum fresh frozen at −80 °C. Within one year serum was tested for antibodies using plasmid transfected HEK293 cells (Autoimmune-Encephalitis-Mosaik1 CA 1439–2, Euroimmun, Lübeck, Germany). The transfected cells ectopically expressed either the CASPR2, the N-methyl-D-aspartic acid receptor (NMDAR), the α-amino-3-hydroxy-5-methyl-4-isoxazolepropionic acid receptor (AMPAR), the Leucin-rich glioma inactivated protein (LGI1), or the gamma-aminobutyric acid receptor (GABAB1/B2). Patient serum was diluted 1:10 and incubated with the HEK cells on cover slides. Bound antibodies were labelled with secondary goat anti-human fluorescein-conjugated antibodies (Euroimmun, Lübeck, Germany). Serum from CASPR2 and NMDAR positive patients was used as positive control.

To allow detection of antibodies with an unknown target antigen, biochip mosaics containing hippocampal sections (rat), cerebellum (monkey), and non-transfected EU90 cells (negative control) were used (IIF: Glutamate-Receptor-Mosaic 3, FA Euroimmun, Lübeck, Germany). These were incubated with diluted patient sera for 30 min, washed and labelled using fluorescein isothiocyanate (FITC) anti-human IgG as secondary antibody. Furthermore, diluted patient sera (1:10) were incubated with tissue sections from cerebellum (monkey), washed with secondary anti-human IgG antibodies, and visualization was subsequently performed using 3 3′-diaminobenzidine (DAB) (Vector Laboratories, Burlingame, USA).

A commercially available recombinant line assay was used to test for antibodies against intracellular antigens: Glutamate-Decarboxylase (GAD), Zinc-Finger Protein 4 (Zic4), Delta/Notch-like Epidermal Growth Factor-Related Receptor (DNER), Glia-Nuclear (SOX1), Paraneoplastic Antigen 1 (Ma1), Paraneoplastic Antigen 2 (Ma2), Amphiphysin, Collapsin-Response-Mediator Protein 5 (CRMP5/CV2), Purkinje cell Antigen1 (Yo), Neuronal-Nuclear Antigen1 (HuD), Neuronal-Nuclear Antigen 2 (Ri)(ravo PNS 11 Line Assay, ravo Diagnostika, Freiburg, Germany).

For all experiments, antibody binding was assessed by two independent investigators on an Olympus BX61 microscope. GraphPad Prism version 5.04 was used for statistical evaluation.

### Results

**Fig. 1:**
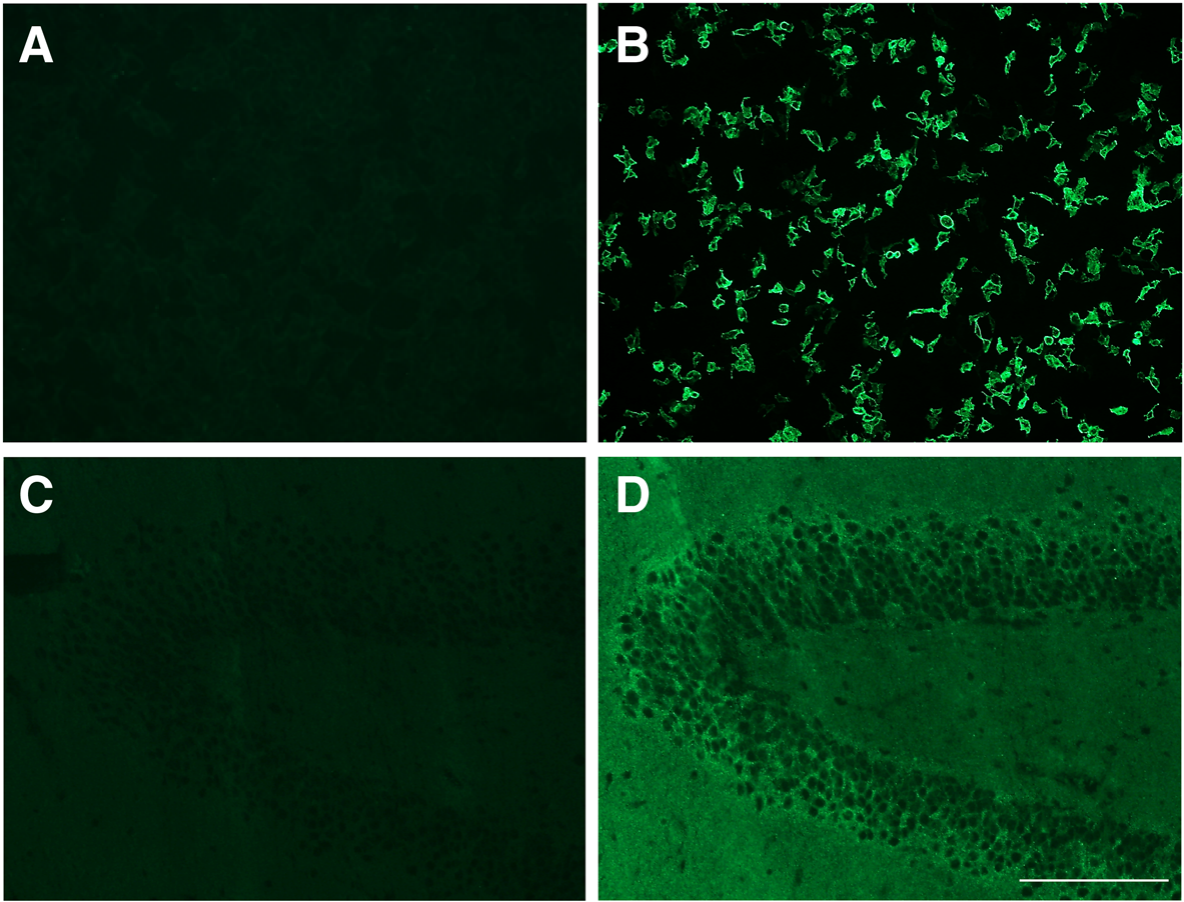
Transfected HEK cells and hippocampus sections - immunofluorescence. **a** transfected HEK cells expressing CASPR2, with patients serum depicting no antibody binding, or positive CASPR2 control **b**, magnification 200fold IIF, scale bar 200 μm. **c** Tissue sections of hippocampus (rat) with patient serum and positive CASPR2 control **d**, magnification 200fold, IIF, scale bar 200 μm

Serum samples from 51 GTS patients between 18 and 64 years (age 35.0 ± 13.1 y, mean ± SD, male/female ratio 3.4:1) were investigated. Using a cell-based assay with transfected HEK293 cells, in none of the 51 patients antibodies against CASPR2 could be detected (see Fig. 1).

Using other specific cell-based assays, we found no evidence for autoantibodies against NMDAR, AMPA1, AMPA2, LGI1, or GABAB1/2 as well. As a screening test for other unspecific neuronal autoantibodies, all sera were incubated with rat tissue sections from cerebellum and hippocampus as well as with monkey cerebellar tissue sections. Visualization was performed either by immunofluorescence or enzyme-linked using DAB to control unspecific background staining. A specific (e.g. anti-neuronal) binding pattern could be found in none of the tissue sections. An anti-nuclear pattern of immunoreactivity was observed in tissue sections in 7/51 (14 %) samples. In these 7 patients, in addition, an immunoblot analysis was performed to investigate antibodies directed against well-defined intracellular target antigens. Yet in none of these patients antibodies against these diagnostically relevant antigens could be detected as summarized in Table 1.

**Table 1:** Method of antibody detection and investigated target antigens

Cell based (HEK293)			Tissue section		Immunblot	
Surface antigen			Site (spezies)			
	Positive	Total		Staining		
CASPR	0	51	hippocamcus (rat)	IIF	GAD65	Amphyphysin
Ampa 1	0	51	cerebellum (rat)	IIF	Zic4	CV2 (CRMP5)
Ampa 2	0	51	cerebellum (monkey)	DAB	Tr (DNER)	Ri
LGI 1	0	51			SOX1	Yo
GABAB 1/2	0	51			Ma 2	HuD
NMDAR	0	51			Ma 1	
			(*n* = 51)		(*n* = 7)	

Transfected cells ectopically expressing (CASPR2), N-methyl-D-aspartic acid receptor (NMDAR), α-amino-3-hydroxy-5-methyl-4-isoxazolepropionic acid receptor (AMPAR), Leucin-rich glioma inactivated protein (LGI1), or gamma-aminobutyric acid receptor (GABAB1/B2). Intracellular antigens: Glutamate-Decarboxylase (GAD), Zinc-Finger-Protein4 (Zic4), Delta/Notch-like Epidermal Growth Factor-Related Receptor (DNER), Glia-Nuclear (SOX1), Paraneoplastic Antigen1 (Ma1), Paraneoplastic Antigen2 (Ma2), Amphiphysin, Collapsin-Response Mediator -Protein 5 (CRMP5/CV2), Purkinje cell Antigen1 (Yo), Neuronal-Nuclear Antigen1 (HuD), Neuronal-Nuclear Antigen2 (Ri)

### Discussion

So far, there is a controversial discussion, whether autoantibodies are involved in the pathophysiology of GTS. While several studies support this “antibody hypothesis” [12, 13], others do not [14, 15]. Substantial evidence for the antibody-hypothesis comes from findings demonstrating positive CSF OCBs in 38 % of patients indicating intrathecal immunoglobulin synthesis in GTS [3]. In addition, morphological alterations further support this assumption because volume increase of e.g. hippocampus in childhood and volume reduction in adulthood resemble inflammatory processes [16]. So far, several studies have been performed investigating anti-streptococcal antibodies and other specific (aldolase C, neuron-specific enolase, non-neuronal enolase and pyruvate kinase M1) [17] and unspecific anti-neuronal or anti-nuclear antibodies [4]. Despite the different autoantibodies of focus, some of the discrepancies might arise from the techniques used, too. For example, the homogenization of tissue necessary for western blotting, might alter protein structure. On the other hand using tissue sections with IIF a specific intracellular antigen might not be available for antibody binding [4] and different serum dilutions cause variable background staining, at the expense of specificity [14]. Using live differentiated neuronal cells or transfected cell lines most of these methodical drawbacks are mitigated and elevated concentrations of anti-neuronal antibodies have been promoted to separate Sydenham chorea from GTS [18].

In this study, we did not detect CASPR2 antibodies, other well-defined neuronal surface antibodies or unspecific anti-neuronal antibodies in sera of patients with GTS. In tissue sections an anti-nuclear pattern of immunoreactivity was observed in 7 (14 %) of the 51 patients samples, which is in the range of anti-nuclear antibodies in healthy individuals (12–16 %) [19]. In these patients the immunoblot analysis did not reveal any of the diagnostically relevant anti-neuronal antibodies. Therefore our results do not support the hypothesis that anti-neuronal antibodies in serum are an immunopathogenic factor in GTS. This statement is restricted by the assumption that target antigens presented solely in basal ganglia cells or cortical neurons might have been missed.

To promote an autoantibody as immunopathogenic, localization on target structures, disease induction after passive transfer and remission of symptoms after withdrawal are required conditions [20]. Diametrical results have been found in animal experiments as some groups were able to generate tic like symptoms in rats via anti-neuronal antibody transfer from GTS patients [21], while others failed to reproduce these results [22]. In order to be able to prove pathogenicity, it is therefore desirable to look for a specific antibody in a disease like GTS.

The appreciable frequency of CASPR2 antibody seroprevalence is 0.9 % in the general population [23]. Based on a known association of GTS and CNTNAP2 gene disruption which codes for CASPR2 and the role of this antibody in the hyperexcitability in Morvan’s disease, we used a cell-based assay to specifically look for antibodies against this protein. The cell-based assay provides the lowest chance of false results since on the one hand epitope structures are unaltered and on the other hand they are presented on the cell surface. However in sera of patients with GTS, we failed to detect CASPR2 antibodies.

A limitation of this study is that we investigated sera, but not CSF to detect autoantibodies in GTS. Some autoantibodies are detected only in the CSF, but not in serum thus, like in many other psychiatric and neurological diseases CSF investigations would be favorable [24]. However, so far, CASPR2 positivity only in the CSF, but not in serum has not yet been described. Particular advantages are: 1) this is a large, prospective, and hypothesis driven study, combining cell-based assays and tissue with different detection methods and 2) frozen serum samples were processed within one year.

In conclusion, neither serum CASPR2 antibodies nor several other serum neuronal surface antigens such as NMDAR play a role in the pathogenesis of GTS.

## Abbreviations

GTS: Gilles de la Tourette syndrome
CNTNAP2: Contactin-associated protein 2 gene
CASPR2: Contactin-associated protein 2
OCB: Oligoclonal bands
NMDAR: N-methyl-D-aspartic acid receptor
AMPAR: α-amino-3-hydroxy-5-methyl-4-isoxazolepropionic acid receptor
AMPA 1 / AMPA 2: AMPA receptor antibodies
LGI1: Leucin-rich glioma inactivated protein
GABAB1/B2: Gamma-aminobutyric acid receptor
GAD: Glutamate-Decarboxylase
Zic4: Zinc-Finger-Protein4
DNER: Delta/Notch-like Epidermal Growth Factor-Related Receptor
SOX1: Glia-Nuclear
Ma1: Paraneoplastic Antigen1
Ma2: Paraneoplastic Antigen2
CRMP5/CV2: Amphiphysin, Collapsin-Response Mediator -Protein 5
Yo: Purkinje cell Antigen1
HuD: Neuronal-Nuclear Antigen1
Ri: Neuronal-Nuclear Antigen2
TNF-α: Tumor necrosis factor alpha
IL-12: Interleukin 12
CSF: Cerebrospinal fluid
ADHD: Attention Deficit Hyperactivity Disorder
OCD: Obsessive–compulsive disorder
VGKC: Voltage gated potassium channels
FITC: Fluorescein isothiocyanate
DAB: 3 3′-diaminobenzidine
IFF: Immunofluorescence
